# Timing of antibiotic treatment among infected patients with- and without fever - a prospective cohort study in a medical emergency department

**DOI:** 10.1186/1757-7241-21-S2-A47

**Published:** 2013-09-09

**Authors:** Daniel Pilsgaard Henriksen, Christian Borbjerg Laursen, Annmarie Touborg Lassen

**Affiliations:** 1 Medical Emergency Department, Odense University Hospital, Denmark; 2 Department of Respiratory Diseases, Odense University Hospital, Denmark

## Background

Infection is one of the most frequent causes of medical admissions to the emergency department in the adult population. However not all infected patients present with fever (>38º Celsius). As early administration of antibiotic is related to improved prognosis, the aim of the study was to compare the “door-to-antibiotics” time in infected patients admitted to the medical emergency ward with- and without fever at arrival.

## Methods

Prospective observational cohort study of all patients admitted acutely to the medical emergency ward, Odense University Hospital (September 1st 2010 – August 31st 2011). At arrival all patients had their temperature measured (rectal). After discharge, all patient records were evaluated manually and patients with infection were identified due to the National Healthcare Safety Network criteria of infections in combination with clinical judgment where focus was clinically evident. Time of antibiotic treatment was extracted electronically from the electronic patient journal.

We included all adult (≥15 years of age) patients with a first time admission of community-acquired infection within the inclusion period with a registration of antibiotics within the first 24 hours after admission (ATC: J01*).

## Results

There were 8133 admissions in 6257 different patients. 1987 patients fulfilled the inclusion criteria. 1003/2003 (50.5%) presented with fever and 984 (49.5%) without fever. Median age for patients with fever was 69.9 years (range 15.1-99.3 years) and without fever 76.0 years (range 15.0-101.8 years, p<0.0001). Patients with fever more often were male (50.4% vs. 43.9%, p=0.004), but less often had severe comorbidity (Charlson index >2 38.7% vs. 46.3%, p=0.001) than among patients without fever.

In patients with fever, the median time to antibiotic administration was 4.1 hours (IQR 2.5 - 6.3 hours) compared with patients without fever 5.9 hours (IQR: 3.7 – 9.3 hours) p<0.0001. For patients with pulmonary focus the time to antibiotics were 4.0 vs. 5.8 hours, p<0.0001 in patients with and without fever, urinary focus 4.0 vs. 6.7 hours, p<0.0001 and abdominal focus 5.2 vs. 7.8, p=0.004.

## Conclusion

Infected patients with fever have antibiotics administered earlier than infected patients without fever. Infected patients with fever are younger and have less severe comorbidity than infected patients who present without fever.

